# HETEROGENEITY OF AGING IN HUMAN POPULATIONS

**DOI:** 10.1093/geroni/igz038.870

**Published:** 2019-11-08

**Authors:** Jing-Dong Jackie Han

**Affiliations:** CAS-MPG Partner Institute for Computational Biology, Shanghai, China, China

## Abstract

Recently by analyzing the 3D facial images, we generated the first comprehensive mapping of the aging human facial phenome. We constructed a robust age predictor and found that on average people of the same chronological age differ by +/-6 years in facial age, with the deviations increasing after age 40. Using this predictor we identified slow- and fast-agers that are significantly supported by health indicators. We further profiled blood cell mRNA and lncRNA expression by RNA-seq of this cohort and computationally predict their regulatory networks and their contributions to the variation in aging rate among different individuals, and those that are modifiable by their lifestyles. By extending the study to a large Northern Chinese cohort of 10,000 people we can now use deep learning AI approaches to precisely estimate aging status based on 3D facial images and their associations with individuals’ health and medical history.

